# Supplementary material for: COZOID: contact zone identifier for visual analysis of protein-protein interactions
Source: BMC Bioinformatics. 2018 Apr 6;19:125. doi: 10.1186/s12859-018-2113-6 (PMC5889581; doi:10.1186/s12859-018-2113-6)
Supplement: Supplementary file 2 — Software build. Executable binary file of the software tool. (ZIP 118,784 kb) [file 12859_2018_2113_MOESM2_ESM.zip › cozoid/java/docs/beansbinding-1.2.1-doc.zip!/org/jdesktop/el/impl/lang/EvaluationContext.html]

EvaluationContext (Beans Binding)


---


|  |  |  |
| --- | --- | --- |
| **Overview** \| **Package** \| **Class** \| **Use** \| **Tree** \| **Deprecated** \| **Index** \| **Help** | |  |
| **PREV CLASS**   **NEXT CLASS** | **FRAMES**    **NO FRAMES**     **All Classes** |  |
| SUMMARY: NESTED \| FIELD \| CONSTR \| METHOD | DETAIL: FIELD \| CONSTR \| METHOD |  |


---


## org.jdesktop.el.impl.lang Class EvaluationContext

```
java.lang.Object
  org.jdesktop.el.ELContext
      org.jdesktop.el.impl.lang.EvaluationContext
```

---

``` public final class EvaluationContext extends ELContext ```

---

**Field Summary**

**Fields inherited from class org.jdesktop.el.ELContext**

`UNRESOLVABLE_RESULT`


**Constructor Summary**

`EvaluationContext(ELContext elContext,
FunctionMapper fnMapper,
VariableMapper varMapper,
Expression expression)`

`EvaluationContext(ELContext elContext,
FunctionMapper fnMapper,
VariableMapper varMapper,
Expression expression,
boolean trackResolvedProperties)`


| **Method Summary** | |
| --- | --- |
| `void` | `clearResolvedProperties()` |
| `java.lang.Object` | `getContext(java.lang.Class key)`             Returns the context object associated with the given key. |
| `ELContext` | `getELContext()` |
| `ELResolver` | `getELResolver()`             Retrieves the `ELResolver` associated with this context. |
| `Expression` | `getExpression()` |
| `FunctionMapper` | `getFunctionMapper()`             Retrieves the `FunctionMapper` associated with this `ELContext`. |
| `java.util.List<Expression.ResolvedProperty>` | `getResolvedProperties()` |
| `VariableMapper` | `getVariableMapper()`             Retrieves the `VariableMapper` associated with this `ELContext`. |
| `boolean` | `isPropertyResolved()`             Returns whether an `ELResolver` has successfully resolved a given (base, property) pair. |
| `void` | `putContext(java.lang.Class key, java.lang.Object contextObject)`             Associates a context object with this `ELContext`. |
| `void` | `resolvedIdentifier(java.lang.Object base, java.lang.Object property)` |
| `void` | `resolvedProperty(java.lang.Object base, java.lang.Object property)` |
| `void` | `setPropertyResolved(boolean resolved)`             Called to indicate that a `ELResolver` has successfully resolved a given (base, property) pair. |

**Methods inherited from class org.jdesktop.el.ELContext**

`getLocale, setLocale`

**Methods inherited from class java.lang.Object**

`clone, equals, finalize, getClass, hashCode, notify, notifyAll, toString, wait, wait, wait`

**Constructor Detail**

### EvaluationContext

```
public EvaluationContext(ELContext elContext,
                         FunctionMapper fnMapper,
                         VariableMapper varMapper,
                         Expression expression)
```

---


### EvaluationContext

```
public EvaluationContext(ELContext elContext,
                         FunctionMapper fnMapper,
                         VariableMapper varMapper,
                         Expression expression,
                         boolean trackResolvedProperties)
```


**Method Detail**

### getELContext

```
public ELContext getELContext()
```

---


### getFunctionMapper

```
public FunctionMapper getFunctionMapper()
```

:   **Description copied from class: `ELContext`**
:   Retrieves the `FunctionMapper` associated with this
    `ELContext`.

    :   **Specified by:**: `getFunctionMapper` in class `ELContext`
    :   **Returns:**: The function mapper to be consulted for the resolution of EL functions.

---


### getVariableMapper

```
public VariableMapper getVariableMapper()
```

:   **Description copied from class: `ELContext`**
:   Retrieves the `VariableMapper` associated with this
    `ELContext`.

    :   **Specified by:**: `getVariableMapper` in class `ELContext`
    :   **Returns:**: The variable mapper to be consulted for the resolution of EL variables.

---


### getExpression

```
public Expression getExpression()
```

---


### getContext

```
public java.lang.Object getContext(java.lang.Class key)
```

:   **Description copied from class: `ELContext`**
:   Returns the context object associated with the given key.

    The `ELContext` maintains a collection of context objects
    relevant to the evaluation of an expression. These context objects
    are used by `ELResolver`s. This method is used to
    retrieve the context with the given key from the collection.

    By convention, the object returned will be of the type specified by
    the `key`. However, this is not required and the key is
    used strictly as a unique identifier.

    :   **Overrides:**: `getContext` in class `ELContext`
    :   **Parameters:**: `key` - The unique identifier that was used to associate the context object with this `ELContext`. **Returns:**: The context object associated with the given key, or null if no such context was found.

---


### getELResolver

```
public ELResolver getELResolver()
```

:   **Description copied from class: `ELContext`**
:   Retrieves the `ELResolver` associated with this context.

    The `ELContext` maintains a reference to the
    `ELResolver` that will be consulted to resolve variables
    and properties during an expression evaluation. This method
    retrieves the reference to the resolver.

    Once an `ELContext` is constructed, the reference to the
    `ELResolver` associated with the context cannot be changed.

    :   **Specified by:**: `getELResolver` in class `ELContext`
    :   **Returns:**: The resolver to be consulted for variable and property resolution during expression evaluation.

---


### isPropertyResolved

```
public boolean isPropertyResolved()
```

:   **Description copied from class: `ELContext`**
:   Returns whether an `ELResolver` has successfully resolved a
    given (base, property) pair.

    The `CompositeELResolver` checks this property to determine
    whether it should consider or skip other component resolvers.

    :   **Overrides:**: `isPropertyResolved` in class `ELContext`
    :   **Returns:**: true if the property has been resolved, or false if not. **See Also:**: `CompositeELResolver`

---


### putContext

```
public void putContext(java.lang.Class key,
                       java.lang.Object contextObject)
```

:   **Description copied from class: `ELContext`**
:   Associates a context object with this `ELContext`.

    The `ELContext` maintains a collection of context objects
    relevant to the evaluation of an expression. These context objects
    are used by `ELResolver`s. This method is used to
    add a context object to that collection.

    By convention, the `contextObject` will be of the
    type specified by the `key`. However, this is not
    required and the key is used strictly as a unique identifier.

    :   **Overrides:**: `putContext` in class `ELContext`
    :   **Parameters:**: `key` - The key used by an @{link ELResolver} to identify this context object.: `contextObject` - The context object to add to the collection.

---


### setPropertyResolved

```
public void setPropertyResolved(boolean resolved)
```

:   **Description copied from class: `ELContext`**
:   Called to indicate that a `ELResolver` has successfully
    resolved a given (base, property) pair.

    The `CompositeELResolver` checks this property to determine
    whether it should consider or skip other component resolvers.

    :   **Overrides:**: `setPropertyResolved` in class `ELContext`
    :   **Parameters:**: `resolved` - true if the property has been resolved, or false if not. **See Also:**: `CompositeELResolver`

---


### clearResolvedProperties

```
public void clearResolvedProperties()
```

---


### resolvedIdentifier

```
public void resolvedIdentifier(java.lang.Object base,
                               java.lang.Object property)
```

---


### resolvedProperty

```
public void resolvedProperty(java.lang.Object base,
                             java.lang.Object property)
```

---


### getResolvedProperties

```
public java.util.List<Expression.ResolvedProperty> getResolvedProperties()
```


---


|  |  |  |
| --- | --- | --- |
| **Overview** \| **Package** \| **Class** \| **Use** \| **Tree** \| **Deprecated** \| **Index** \| **Help** | |  |
| **PREV CLASS**   **NEXT CLASS** | **FRAMES**    **NO FRAMES**     **All Classes** |  |
| SUMMARY: NESTED \| FIELD \| CONSTR \| METHOD | DETAIL: FIELD \| CONSTR \| METHOD |  |


---
